# PpCBF6 Is Involved in Phytosulfokine α-Retarded Chilling Injury by Suppressing the Expression of *PpLOX5* in Peach Fruit

**DOI:** 10.3389/fpls.2022.874338

**Published:** 2022-04-29

**Authors:** Caifeng Jiao

**Affiliations:** School of Horticulture, Anhui Agricultural University, Hefei, China

**Keywords:** phytosulfokine α, chilling injury, lipoxygenase 5, PpCBF6, peaches

## Abstract

The involvement of PpCBF6 in phytosulfokine α (PSKα)-ameliorated chilling injury (CI) by suppressing the expression of lipoxygenase 5 (LOX5) in peach fruit was revealed. The peaches were immersed in distilled water and PSKα solution. PSKα application inhibited the progression of CI index and weight loss, and the reduction of firmness and total soluble solids content in peaches. The endogenous PSKα accumulation and gene expression of PSK receptor 1 (*PSKR1*) and *PSKR2* were up regulated by PSKα application. The superoxide anion (O_2_^–^) production rate, hydrogen peroxide (H_2_O_2_) production and reactive oxygen species (ROS) content decreased by PSKα application. Furthermore, PSKα application reduced the gene expression of 12 *PpLOXs* and LOX activity. The gene expression of 6 *PpCBFs* was enhanced by PSKα application. Importantly, after PSKα application, among 12 *PpLOXs*, the decrease in gene expression of *PpLOX5* was the lowest, and among 6 *PpCBFs*, the increase in gene expression of *PpCBF6* was the highest. Further results suggested that PpCBF6 bound to the C-repeat/dehydration responsive element (CRT/DRE) motif in *PpLOX5* promoter, and repressed its transcription. Thus, PpCBF6 was involved in the PSKα-retarded CI by inhibiting the expression of *PpLOX5* in peaches.

## Introduction

Refrigeration is commonly employed to maintain fruit quality and to extend storage period (Belay et al., 2020). However, peaches are susceptible to chilling injury (CI) when subjected to cold stress (Nilo et al., 2010). The main CI symptom in peaches is internal browning (Chen et al., 2019), which causes certain economic losses. Thus, it is vital to hunt for potent approaches to relieve CI in peaches.

Phytosulfokine α (PSKα), a plant sulfonated pentapeptide growth regulator, has been verified to motivate many biochemical activities (Aghdam and Luo, 2021a). PSKα perception by leucine-rich repeat PSK receptor (PSKR) kinase in plasma membrane is vital for triggering a series of physiological reactions (Aghdam et al., 2021b). Accordingly, PSKα application promoted energy status and scavenged reactive oxygen species (ROS) overproduction, and thus retarded senescence in broccoli florets throughout cold storage (Aghdam and Luo, 2021b). PSKα treatment boosted cyclic guanosine monophosphate accumulation and ameliorated membrane damage, therefore delaying petals senescence and extending vase life of cut rose flowers (Aghdam et al., 2021a). PSKα treatment retarded senescence in strawberries by inducing the phenylpropanoid pathway throughout cold storage (Aghdam et al., 2021b). However, whether PSKα treatment could relieve CI in peaches remains to be explored.

Cold storage would result in the elevation of lipoxygenase (LOX) level in postharvest fruit (Sheng et al., 2016). LOX catalyzes the hydroperoxidation of unsaturated fatty acids to produce hydroperoxide and a lot of ROS, leading to cell membrane damage and browning. Therefore, to inhibit CI in cold sensitive fruit, it is critical to apply potent measures to reduce LOX level. Accordingly, PSKα treatment could retard the increase in gene expression and activity of LOX in broccoli florets (Aghdam and Flores, 2021c), indicating that PSKα application is an effective technology to relieve cell membrane damage in postharvest vegetable. However, the effects of PSKα treatment on LOX level in peaches remain to be revealed.

Besides, the motivation of transcriptional factors (TFs) is an important defense response to cold stress in postharvest fruit (Peng et al., 2020; Jiao, 2021). C-repeat binding factors (CBFs) are the most widely investigated cold resistance TFs (Xiao et al., 2010; Liang et al., 2013). Transcription level of CBF1 in tomatoes was positively correlated with chilling tolerance, and negatively correlated with CI severity, suggesting that CBFs can effectively reflect chilling tolerance in fruit (Arae et al., 2017). Moreover, CBFs could be activated by lots of exogenous applications in plants. Methyl jasmonate treatment promoted the gene expression of *CBF6*, and thus enhanced the chilling tolerance in peaches (Cao et al., 2021). NO treatment enhanced the tolerance to cold stress by up regulating *CBF1* expression in kiwifruit (Jiao, 2021). However, the modulation of CBFs by PSKα treatment remains to be explored.

Moreover, CBFs modulate the expression of downstream genes by binding to the C-repeat/dehydration responsive element (CRT/DRE) motif (CCGAC) in their promoters (Cao et al., 2021). Accordingly, CBF3 bound to the CRT/DRE motif in the promoter of ureidoglycolate amidohydrolase, and enhanced its expression in rice (Li et al., 2015). CBF6 suppressed the expression of vacuolar invertase by interacting with the CRT/DRE-binding site in its promoter, and thus retarded CI in peaches (Cao et al., 2021). However, the regulation of LOXs by CBFs in peaches remains to be investigated.

The aims of this research were to explore the effects of PSKα application on the decrease in CI severity and the induction of PpLOX5 and PpCBF6, and the modulation of *PpLOX5* promoter by PpCBF6 in peaches.

## Materials and Methods

### Plant Material and Postharvest Applications

Peaches (*Prunus persica* Batsch cv. ‘Yuhua No. 3’) were obtained at 80% ripeness from the orchard in Nanjing, China. The chosen uniform peaches absence of visual defects were divided into two groups each of three biological replicates at random. For each biological replicate of each group, 400 fruit were used.

(1) Control (CK): The peaches were immersed in distilled water.
(2) PSKα: The peaches were immersed in 300 nM PSKα.

The PSKα concentration was determined according to my preliminary experiments (Supplementary Figure 1). The aforementioned fruit were immersed for 10 min and air dried for 40 min thereafter. The peaches were stored at 4 ± 1°C for 35 days under 80–90% relative humidity afterward. For each biological replicate, 30 peaches were used to determine the CI degree, weight loss, firmness, and total soluble solids content every 7 days. For each biological replicate, 20 peaches were used to detect the physiological indicators (O_2_^–^ production rate, H_2_O_2_ production, ROS content, LOX activity, and gene expression of *PpLOXs* and *PpCBFs*) every 7 days, which were preserved at −80°C. For CI severity calculation, peaches were removed to 20°C for 3 days after each time point. Other indicators were evaluated immediately after each time point.

### Chilling Injury Severity, Weight Loss, Firmness, and Total Soluble Solids Content Calculation

For CI index calculation, the severity of internal browning in each fruit was recorded: 0 = none, 1 ≤ 5%, 2 = 6–25%, 3 = 26–50%, 4 ≥ 50%. CI index = Σ (CI severity × number of peaches at the CI severity)/(5 × total number of peaches in the replicate).

For weight loss assay, the peaches were weighed before and after each time point.

Firmness was determined using the firmness analyzer (FT327, Effegi, Alfonsine, Italy) with a probe of a 7.5 mm penetration depth.

For total soluble solids content determination, 5.0 g peach samples were ground, and centrifuged at 9,000 *g* for 15 min. The collected supernatant was assayed using WYT-4 hand-held refractometer (Shanghai Precision & Scientific Instrument Co., Ltd., Shanghai, China) afterward.

### Endogenous PSKα Accumulation Assay

Five gram of peach samples were incubated with 8 M urea for 60 min at 4°C as described by Song et al. (2017). After pH was adjusted to 2.0–3.0 using 1 M HCl, the homogenate was incubated for 15 min at 4°C, followed by a centrifugation at 12,000 *g* for 30 min at 4°C. The endogenous PSKα accumulation was detected using the protocol of Aghdam et al. (2021b).

### O_2_^–^ Production Rate, H_2_O_2_ Production, and ROS Content Assay

For O_2_^–^ production rate detection, 5.0 g peach samples were homogenized in 50 mM phosphate buffer (pH 7.8), followed by a centrifugation at 11,000 *g* for 20 min at 4°C thxereafter. The O_2_^–^ production rate was measured following the protocol of Wang et al. (2020). The data were expressed as mmol/kg on a fresh weight (FW) basis.

For H_2_O_2_ production measurement, 5.0 g peach samples were homogenized using cold acetone, followed by a centrifugation at 11,000 *g* for 20 min at 4°C thereafter. The H_2_O_2_ production was assayed using the protocol of Yang et al. (2016). The data were expressed as μmol/kg on a FW basis.

The ROS content was assayed using fluorescence spectrophotometer (Cary Eclipse, VARIAN, United States) using the protocol of Jing et al. (2016). The maximum excitation and emission wavelengths were 485 and 530 nm, respectively. The slit width was 5 nm. The data were expressed as a.u./mg on a FW basis.

### Transcriptomic Analysis

The total RNA was extracted according to the protocol of MiniBEST Plant RNA Extraction Kit (Takara Bio Inc., China). After purified, the total RNA was used for cDNA library construction. The clean reads were mapped to peach genome. The read numbers were transformed to FPKM (Fragments Per Kilobase of transcript sequence per Millions base pairs sequenced) value for gene expression quantification. The differentially expressed genes was analyzed using edgeR with following criteria: False discovery rate (FDR) < 0.05 and |log_2_^Fold  change^| ≥ 1. Three biological replicates were included for each assay.

### Lipoxygenase Activity Assay

Five gram of peach samples were extracted in 0.1 M phosphate buffer (pH 6.8) containing 1% (w/v) polyvinylpyrrolidone, and centrifuged at 10,000*g* for 15 min at 4°C thereafter. LOX activity was determined using the protocol of Yao et al. (2019). The data were expressed as U/g on a FW basis.

### Gene Expression Assay

Total RNA in peach samples was acquired following the protocol of E.Z.N.A.™ Plant RNA Kit (Omega, United States). The first-strand cDNA was synthesized using the protocol of Jiao (2021) thereafter. The primers in quantitative real-time polymerase chain reaction (qRT-PCR) tests for *PpPSKR1*, *PpPSKR2*, 12 *PpLOXs, 6 PpCBFs*, and *β-actin* were designed (Table 1). The gene expression was assayed according to the protocol of Jiao (2021). Three replicates were included for each assay.

**TABLE 1:** The primers for qRT-PCR tests.

Gene	Gene ID (LOC)	Primer name	Primer sequences (5′→3′)	Amplicon size (bp)
*PpPSKR1*	18793371	Sense	GGTAACAGGCTTTCGGGGAT	102
		Antisense	CAAATCCAACGGCCATTCCG	
*PpPSKR2*	18779076	Sense	CAACCTGTAGGGGCGATGTT	116
		Antisense	CGAGCCACGAGACAACTTCT	
*PpLOX2-1*	18773995	Sense	ATTCATCCATGGCAGTCGCA	100
		Antisense	CGTCACAGTTATGGTGGCCT	
*PpLOX2-1*	18787524	Sense	AATGCATGGACAATTCTGGCAA	102
		Antisense	CAACCCCGTCTTGGAGTCAA	
*PpLOX2-1*	18787140	Sense	AGAGACCCGAAATGGCACTG	120
		Antisense	AGCCCACCCAATAACCAAAGT	
*PpLOX3-1*	18781374	Sense	TGATGGGACGGGACTCAGAT	123
		Antisense	GGCAAACAACAAGATATAAGCCCA	
*PpLOX3-1*	18783171	Sense	ACAAAAACTGGCGCTTCGAC	110
		Antisense	TCGAGCACAAGTCTCACACC	
*PpLOX6*	18793349	Sense	AGCCCCATCCAGTTTACGTG	109
		Antisense	ACCTTCCGGCTGAGAAAGTG	
*PpLOX6*	18766622	Sense	ACCCTTCCTTGTTAGTCAGCAG	113
		Antisense	TGCATTTTGACTGCCTGTGC	
*PpLOX5*	18774987	Sense	CAACCGTTGACTTCGGCTTC	100
		Antisense	GGGTCACCCTTAACGGAACT	
*PpLOX5*	18773359	Sense	CAGAGAGGCACCCCAGAATG	112
		Antisense	CCATGGTCATGGATGGGCTT	
*PpLOX5*	18774870	Sense	AAAGACCAGAACTTGAGGCCA	104
		Antisense	GTCATGCGCAAGAAACCAGG	
*PpLOX5*	18774983	Sense	CACTCCTGAGTGGACAGCAG	112
		Antisense	TTACAAGGCCTTATTGCAACTGT	
*PpLOX5*	18775056	Sense	TGAAGAACCGAGTTGGACCG	109
		Antisense	ACAAGGCCTTATTGCAACTGT	
*PpCBF1*	18778067	Sense	TTCAAAGAGACGAGGCACCC	113
		Antisense	ACGGAGCACAGTACCAGTCTA	
*PpCBF2*	18776669	Sense	TCGAGTTCTTTCTCCGACGC	100
		Antisense	GTCGGATACGTTCCAAGCCA	
*PpCBF3*	18776409	Sense	AGCCCGAGTCGAGTTCTTTG	103
		Antisense	TTCATGTCGCTGCCTAAGGG	
*PpCBF4*	18776400	Sense	GCTTCTCTGGAAAACCCGGA	108
		Antisense	CTTCGGATAAGTCCCGAGCC	
*PpCBF5*	18777414	Sense	GCCCAAGAAGACGAAGTCCA	112
		Antisense	CGGTTCCCATGTCATCCCAA	
*PpCBF6*	18787317	Sense	CAGGATTTGGCTCGGGACTT	106
		Antisense	TCGGCAAAGTTCAAGCAAGC	
*β-actin*	18779708	Sense	GTTATTCTTCATCGGCGTCTTCG	109
		Antisense	CTTCACCATTCCAGTTCCATTGTC	

### Yeast One-Hybrid Assay

A yeast one-hybrid (Y1H) assay was performed using the protocol of the Clontech ^®^ Matchmaker ^®^ one-hybrid system. The three tandem copies of the CRT/DRE-binding site (CCGAC) (-405 to -401 bp) and adjacent nucleotides in the *PpLOX5* promoter (Supplementary Text 1) were ligated into pAbAi vector. The *PpLOX5*-AbAi and p53-AbAi were introduced into the Y1H Gold strain thereafter. Positive yeast cells were transformed with the pGADT7-AD, which contained the coding sequences (CDS) of PpCBF6. The basal activity of the promoter was detected on the SD medium lacking Ura with Aureobasidin A (AbA). Whether PpCBF6 could bind to *PpLOX5* promoter was judged by the growth status of co-transformants on SD/-Leu medium in the presence of AbA.

### Dual Luciferase Reporter Assay

The sequences of *PpLOX5* promoter were inserted into the pGreen II 0800-LUC vector. The CDS of PpCBF6 were inserted into the pGreen II 62-SK vector. The plasmids were introduced into *Agrobacterium tumefaciens* strain GV3101, and transiently expressed in tobacco thereafter. After 3 days, LUC and REN were determined using the dual luciferase reporter (DLR) assay system (Promega). Nine independent replicates were conducted for each combination. The data were expressed as the relative LUC/REN ratio.

### Statistical Analysis

Statistical analysis was performed using SPSS 22.0 (SPSS Inc., Chicago, IL, United States). Each data was analyzed with one-way analysis of variance. The significant differences at *p* < 0.05 were determined using Duncan test.

## Results

### PSKα Application Reduced the Chilling Injury Degree and Weight Loss and Maintained the Firmness and Total Soluble Solids Content in Peaches

**FIGURE 1:**
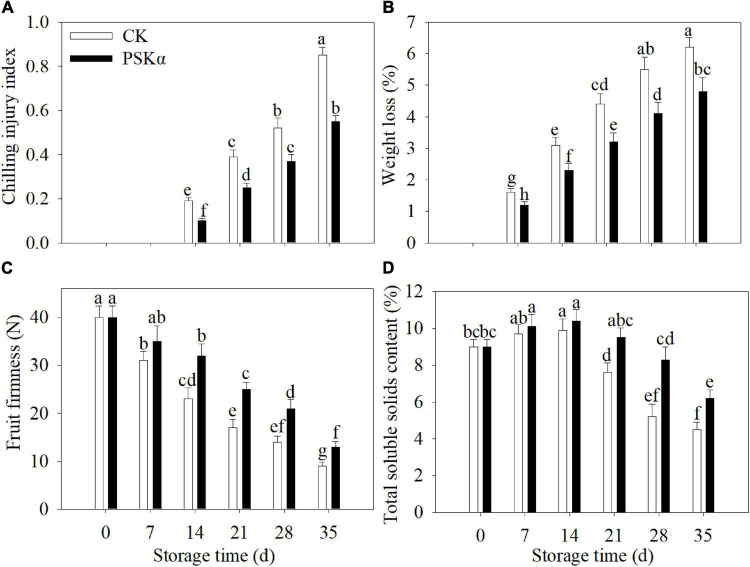
PSKα application reduced the CI degree **(A)** and weight loss **(B)** and maintained the firmness **(C)** and total soluble solids content **(D)** in peaches. The CI degree **(A)** was analyzed after peaches were removed to 20°C for 3 days after each time point. The weight loss **(B)**, firmness **(C)**, and total soluble solids content **(D)** were detected immediately after each time point. For each biological replicate, after each time point, 30 peaches were used to determine the CI degree **(A)** and weight loss **(B)** and maintained the firmness **(C)** and total soluble solids content **(D)**. Values represent the mean ± standard deviation. Values not with the same letter are significantly different at *p* < 0.05.

The CI disorder in peaches was initially found at 14 days. The CI index and weight loss continuously increased following control and PSKα treatments throughout storage. PSKα treatment markedly delayed the increase in CI index and weight loss. Following 35 days of storage, PSKα treatment caused the decrease in CI index and weight loss by 35 and 23% (Figures 1A,B).

The firmness continuously decreased in peaches following control and PSKα treatments throughout storage. PSKα treatment markedly delayed the decrease in firmness. Following 35 days of storage, PSKα treatment boosted the firmness by 44% (Figure 1C).

The total soluble solids content increased firstly and decreased thereafter in peaches following control and PSKα treatments throughout storage. PSKα treatment markedly retarded the decrease in total soluble solids content. Following 35 days of storage, PSKα treatment caused the elevation of total soluble solids content by 38% (Figure 1D).

### PSKα Application Elevated the Endogenous PSKα Accumulation and Gene Expression of *PSKR1* and *PSKR2* in Peaches

**FIGURE 2:**
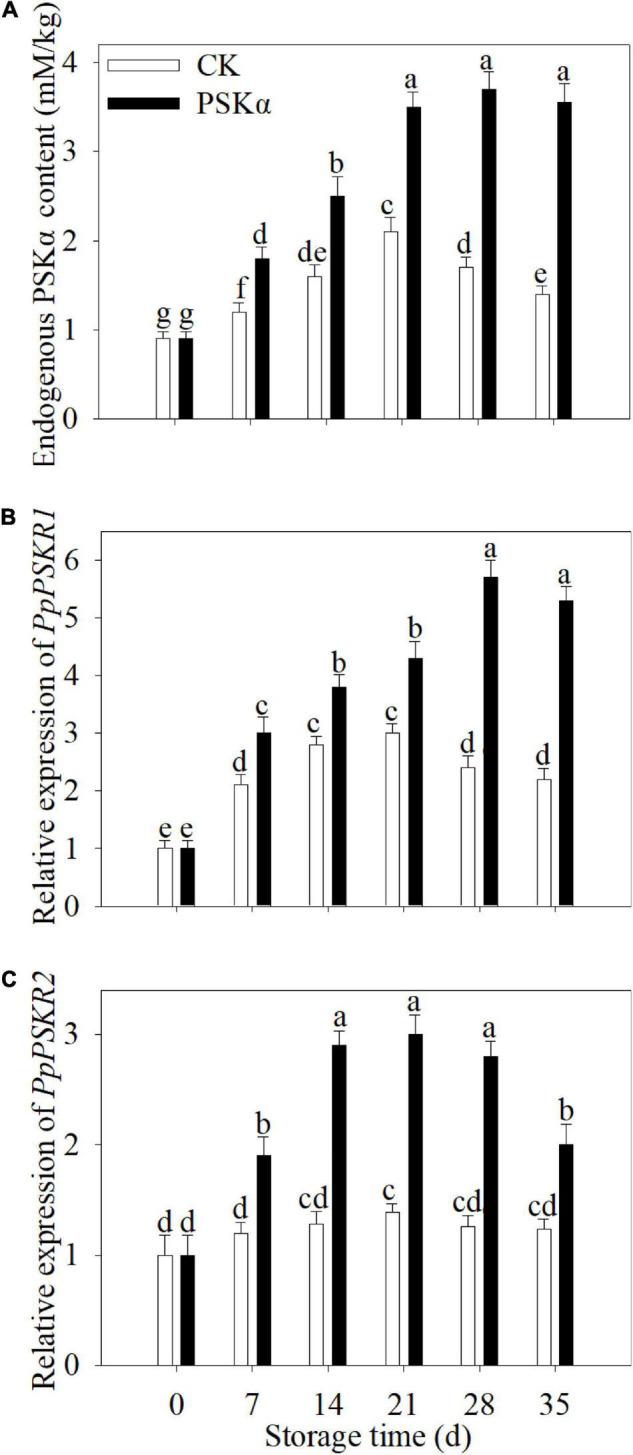
PSKα application elevated the endogenous PSKα accumulation **(A)** and gene expression of *PSKR1* **(B)**, and *PSKR2* **(C)** in peaches. The endogenous PSKα accumulation **(A)** and gene expression of *PSKR1* **(B)** and *PSKR2* **(C)** were determined immediately after each time point. For each biological replicate, after each time point, 20 peaches were used to detect the endogenous PSKα accumulation **(A)** and gene expression of *PSKR1* **(B)** and *PSKR2* **(C)**. Values represent the mean ± standard deviation. Values not with the same letter are significantly different at *p* < 0.05.

The endogenous PSKα accumulation and gene expression of *PSKR1* and *PSKR2* increased firstly and decreased thereafter following control and PSKα applications throughout storage in peaches. PSKα application promoted the endogenous PSKα accumulation and gene expression of *PSKR1* and *PSKR2*. Following 35 d of storage, the endogenous PSKα accumulation and gene expression of *PSKR1* and *PSKR2* in PSKα-immersed peaches was 2.5, 2.4, and 1.6 times of the control (Figure 2).

### PSKα Application Reduced the O_2_^–^ Production Rate, H_2_O_2_ Production and ROS Accumulation in Peaches

**FIGURE 3:**
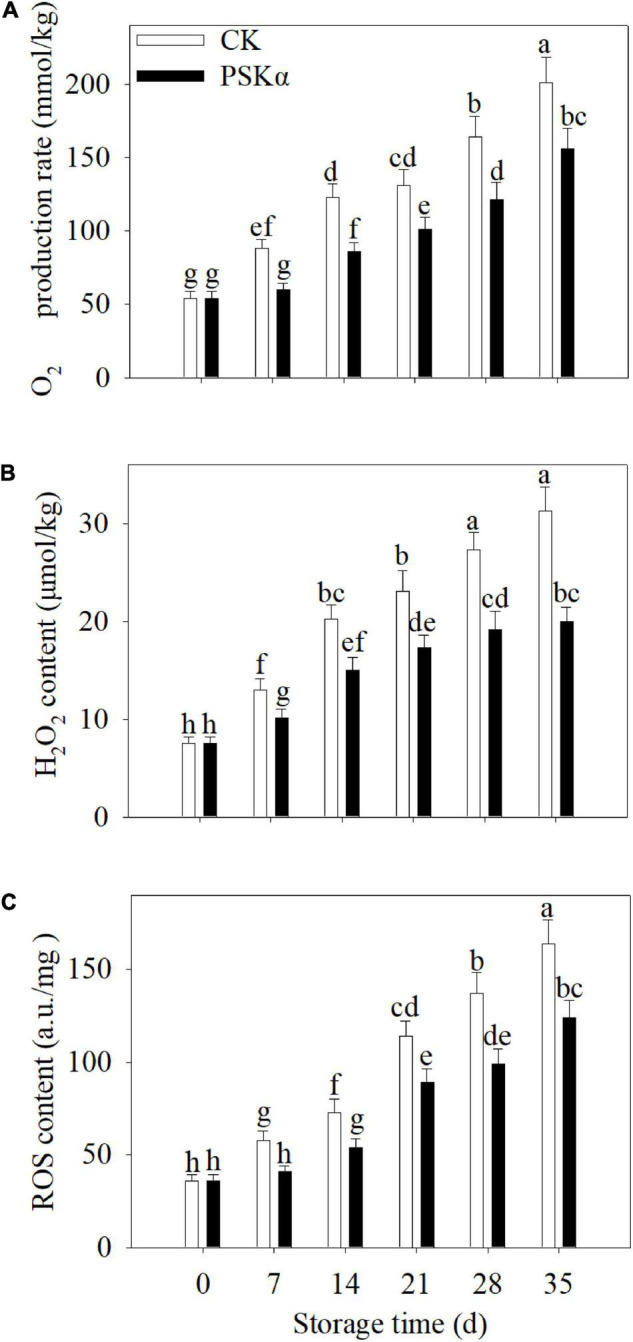
PSKα application retarded the elevation of the O_2_^–^ production rate **(A)**, H_2_O_2_ content **(B)** and ROS accumulation **(C)** in peaches. The O_2_^–^ production rate **(A)**, H_2_O_2_ content **(B)**, and ROS accumulation **(C)** were assayed immediately after each time point. For each biological replicate, after each time point, 20 peaches were used to detect the O_2_^–^ production rate **(A)**, H_2_O_2_ content **(B)**, and ROS accumulation **(C)**. Values represent the mean ± standard deviation. Values not with the same letter are significantly different at *p* < 0.05.

The O_2_^–^ production rate, H_2_O_2_ production and ROS accumulation continuously increased following control and PSKα applications throughout storage in peaches. PSKα application suppressed the elevation of O_2_^⋅–^ production rate, H_2_O_2_ production and ROS content. Following 35 days of storage, the O_2_⋅^−^ production rate, H_2_O_2_ production and ROS accumulation in PSKα-immersed peaches were suppressed by 22, 36, and 24% (Figure 3).

### PSKα Application Reduced the Gene Expression of *PpLOXs* and LOX Activity in Peaches

Twelve *PpLOXs* were identified using transcriptome. Following PSKα application, the gene expression of *PpLOX2-1* (LOC18773995), *PpLOX2-1* (LOC18787524), *PpLOX2-1* (LOC18787140), *PpLOX3-1* (LOC18781374), and *PpLOX3-1* (LOC18783171) showed no significant change. The gene expression of *PpLOX6* (LOC18793349), *PpLOX6* (LOC18766622), *PpLOX5* (LOC18774987), *PpLOX5* (LOC18773359), *PpLOX5* (LOC18774870), *PpLOX5* (LOC18774983), and *PpLOX5* (LOC18775056) decreased after PSKα application (Table 2).

**TABLE 2:** The identification and quantification of *PpLOXs* in postharvest peaches after PSKα treatment using transcriptome.

Gene ID (LOC)	Gene description	Log_2_^Fold*change*^				
		7 days	14 days	21 days	28 days	35 days
18773995	Linoleate 13S-lipoxygenase 2-1	−0.22 ± 0.02	−0.45 ± 0.06	−0.47 ± 0.04	−0.37 ± 0.04	−0.49 ± 0.05
18787524	linoleate 13S-lipoxygenase 2-1	−0.73 ± 0.05	−0.38 ± 0.04	−0.51 ± 0.06	−0.37 ± 0.04	−0.79 ± 0.08
18787140	linoleate 13S-lipoxygenase 2-1	−0.25 ± 0.02	−0.26 ± 0.05	−0.69 ± 0.03	−0.20 ± 0.04	−0.31 ± 0.04
18781374	linoleate 13S-lipoxygenase 3-1	−0.73 ± 0.05	−0.84 ± 0.04	−0.44 ± 0.05	−0.46 ± 0.07	−0.39 ± 0.04
18783171	linoleate 13S-lipoxygenase 3-1	−0.28 ± 0.03	−0.37 ± 0.04	−0.59 ± 0.06	−0.62 ± 0.06	−0.48 ± 0.05
18793349	Lipoxygenase 6	−0.24 ± 0.03	−0.42 ± 0.04	−1.18 ± 0.05	−0.39 ± 0.04	−1.02 ± 0.04
18766622	Linoleate 9S-lipoxygenase 6	−0.83 ± 0.05	−1.12 ± 0.08	−1.25 ± 0.09	−1.35 ± 0.07	−1.27 ± 0.09
18774987	Probable linoleate 9S-lipoxygenase 5	−1.36 ± 0.13	−1.27 ± 0.15	−1.42 ± 0.15	−1.25 ± 0.16	−1.49 ± 0.15
18773359	Probable linoleate 9S-lipoxygenase 5	−1.47 ± 0.14	−1.52 ± 0.20	−1.62 ± 0.15	−0.99 ± 0.09	−1.37 ± 0.16
18774870	Probable linoleate 9S-lipoxygenase 5	−1.99 ± 0.14	−2.26 ± 0.27	−2.27 ± 0.15	−2.69 ± 0.14	−2.37 ± 0.16
18774983	Probable linoleate 9S-lipoxygenase 5	−1.38 ± 0.09	−1.40 ± 0.07	−1.19 ± 0.09	−1.62 ± 0.15	−1.38 ± 0.09
18775056	Probable linoleate 9S-lipoxygenase 5	−1.44 ± 0.08	−1.57 ± 0.19	−1.35 ± 0.16	−1.57 ± 0.09	−1.14 ± 0.11

*|log_2_^Fold  change^| ≥ 1 represents up-regulation, while 0 < |log_2_^Fold  change^| < 1 represents no statistical difference.*

**FIGURE 4:**
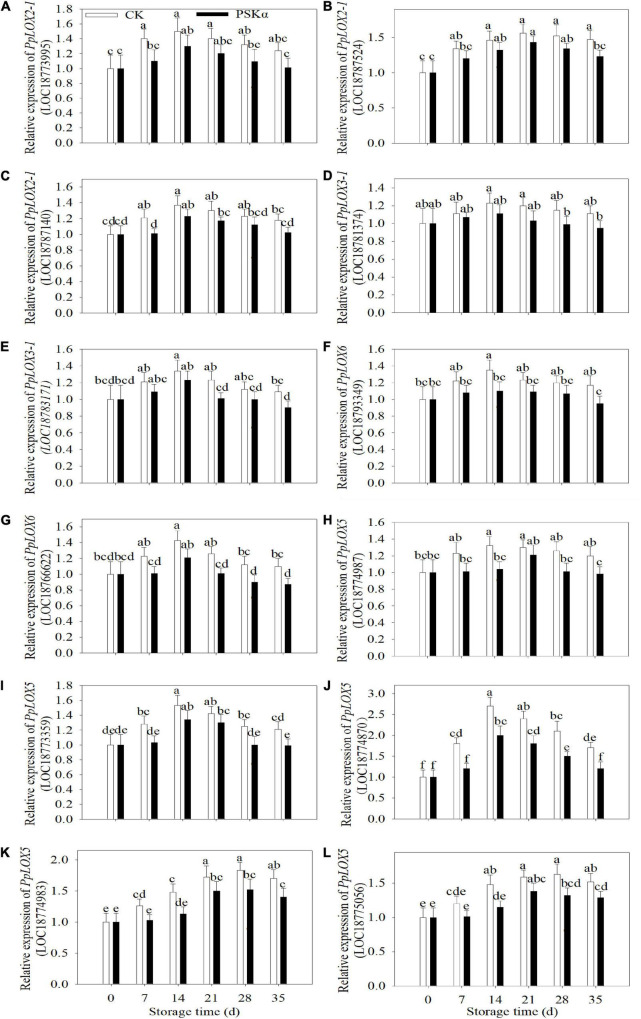
Effects of PSKα application on the gene expression of *PpLOX2-1* (LOC18773995) **(A)**, *PpLOX2-1* (LOC18787524) **(B)**, *PpLOX2-1* (LOC18787140) **(C)**, *PpLOX3-1* (LOC18781374) **(D)**, *PpLOX3-1* (LOC18783171) **(E)**, *PpLOX6* (LOC18793349) **(F)**, *PpLOX6* (LOC18766622) **(G)**, *PpLOX5* (LOC18774987) **(H)**, *PpLOX5* (LOC18773359) **(I)**, *PpLOX5* (LOC18774870) **(J)**, *PpLOX5* (LOC18774983) **(K)**, and *PpLOX5* (LOC18775056) **(L)** in peaches. The gene expression of *PpLOXs* were determined immediately after each time point. For each biological replicate, after each time point, 20 peaches were used to detect the gene expression of *PpLOXs*. Values represent the mean ± standard deviation. Values not with the same letter are significantly different at *p* < 0.05.

To verify the results of transcriptome, qRT-PCR tests were carried out. Following 35 days of storage, there were no statistical differences in the gene expression of *PpLOX2-1* (LOC18773995), *PpLOX2-1* (LOC18787140), and *PpLOX3-1* (LOC18781374) between CK and PSKα treatments. Following 35 days of storage, the gene expression of *PpLOX2-1* (LOC18787524), *PpLOX3-1* (LOC18783171), *PpLOX6* (LOC18793349), *PpLOX6* (LOC18766622), *PpLOX5* (LOC18774987), *PpLOX5* (LOC18773359), *PpLOX5* (LOC18774870), *PpLOX5* (LOC18774983), and *PpLOX5* (LOC18775056) in PSKα-immersed peaches decreased by 16, 17, 19, 21,18, 18, 29, 17, and 15% (Figure 4).

**FIGURE 5:**
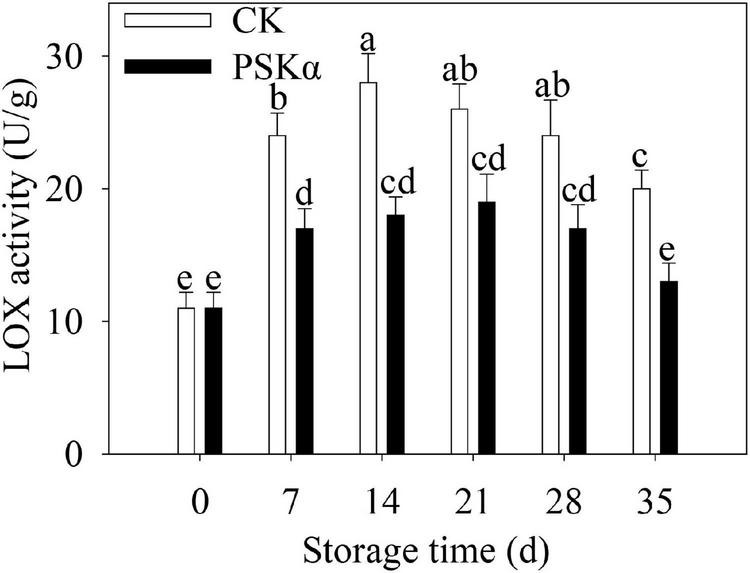
PSKα application suppressed the LOX activity in peaches. The LOX activity were determined immediately after each time point. For each biological replicate, after each time point, 20 peaches were used to detect the LOX activity. Values represent the mean ± standard deviation. Values not with the same letter are significantly different at *p* < 0.05.

The LOX activity increased firstly and decreased thereafter following control and PSKα applications throughout storage in peaches. PSKα treatment suppressed the LOX activity. The LOX activity after PSKα treatment was at the summit on 21 days, which decreased by 27%. Following 35 days of storage, the LOX activity in PSKα-immersed peaches decreased by 35% (Figure 5).

### PSKα Treatment Enhanced the Gene Expression of *PpCBFs* in Peaches

Six *PpCBFs* were identified using transcriptome. During storage, PSKα application enhanced the gene expression of six *PpCBFs* (Table 3).

**TABLE 3:** The identification and quantification of *PpCBF*s in postharvest peaches after PSKα treatment using transcriptome.

Gene ID (LOC)	Gene description	Log_2_^Fold*change*^				
		7 days	14 days	21 days	28 days	35 days
18778067	C-repeat binding factor 1	1.09 ± 0.08	1.35 ± 0.13	1.48 ± 0.18	1.59 ± 0.09	1.28 ± 0.12
18776669	C-repeat binding factor 2	1.29 ± 0.08	1.03 ± 0.13	1.59 ± 0.12	1.73 ± 0.17	1.52 ± 0.06
18776409	C-repeat binding factor 3	1.49 ± 0.05	1.84 ± 0.04	1.15 ± 0.14	1.27 ± 0.14	1.70 ± 0.06
18776400	C-repeat binding factor 4	1.59 ± 0.09	1.48 ± 0.07	1.37 ± 0.08	1.41 ± 0.09	1.18 ± 0.16
18777414	C-repeat binding factor 5	1.31 ± 0.09	1.18 ± 0.17	0.72 ± 0.08	1.19 ± 0.08	1.48 ± 0.18
18787317	C-repeat binding factor 6	2.57 ± 0.15	1.97 ± 0.09	2.25 ± 0.11	2.39 ± 0.16	2.12 ± 0.15

*|log_2_^Fold  change^| ≥ 1 represents up-regulation, while 0 < |log_2_^Fold  change^| < 1 represents no statistical difference.*

**FIGURE 6:**
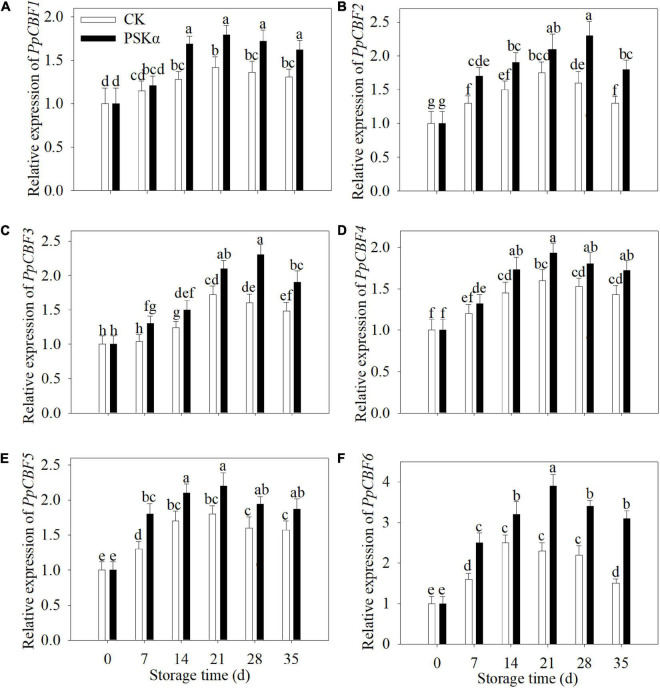
PSKα application enhanced the gene expression of *PpCBF1* **(A)**, *PpCBF2* **(B)**, *PpCBF3* **(C)**, *PpCBF4* **(D)**, *PpCBF5* **(E)**, and *PpCBF6* **(F)** in peaches. The gene expression of *PpCBFs* was detected immediately after each time point. For each biological replicate, after each time point, 20 peaches were used to detect the gene expression of *PpCBFs*. Values represent the mean ± standard deviation. Values not with the same letter are significantly different at *p* < 0.05.

To verify the results of transcriptome, qRT-PCR tests were carried out. Following 35 days of storage, the gene expression of *PpCBF1* (LOC18778067), *PpCBF2* (LOC18776669), *PpCBF3* (LOC18776409), *PpCBF4* (LOC18776400), *PpCBF5* (LOC18777414) and *PpCBF6* (LOC18787317) in PSKα-immersed peaches was 1.2, 1.4, 1.3, 1.2, 1.2, and 2.1 times of the control (Figure 6).

### The Suppression of PpLOX5 Promoter by PpCBF6

**FIGURE 7:**
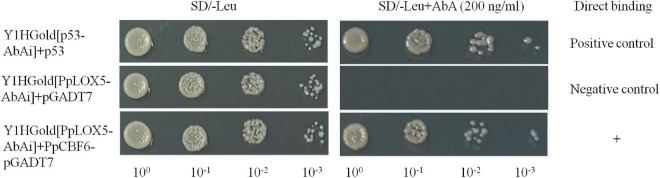
The interaction between PpCBF6 and *PpLOX5* promoter. The direct binding of PpCBF6 protein to *PpLOX5* promoter was tested on the basis of the ability of Y1HGold [*PpLOX5*-AbAi] + PpCBF6-pGADT7 to grow on SD/-Leu in the presence of 200 ng/ml AbA.

The promoter sequences of *PpLOX5* were characterized, and a putative CRT/DRE-binding site (CCGAC) was identified (Supplementary Text 1). Then, Y1H assay was performed to explore the interaction between PpCBF6 and *PpLOX5* promoter. the results in Y1H assay showed that yeast cells co-transformed with pGADT7-PpCBF6 and pAbAi-*PpLOX5* promoter grew in the presence of 200 ng/ml AbA, indicating that PpCBF6 bound to the CRT/DRE motif in the *PpLOX5* promoter (Figure 7).

**FIGURE 8:**
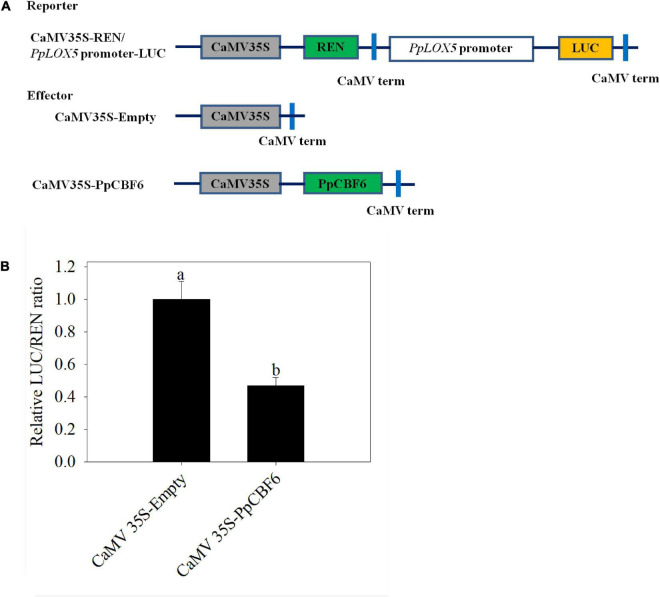
The regulation of *PpLOX5* promoter by PpCBF6. **(A)** Schematic of the reporter and effecter constructs. **(B)** The inhibition of *PpLOX5* promoter by PpCBF6 protein. Nine independent replicates were included for each combination. Transactivation was indicated by the LUC/REN ratio. Values represent the mean ± standard deviation. Values not with the same letter are significantly different at *p* < 0.05.

Furthermore, as indicated from the DLR assay, compared with the control that was cotransfected with the empty vector, the relative LUC/REN ratio decreased when the promoter-LUC reporter construct was cotransfected with PpCBF6, suggesting that PpCBF6 suppressed *PpLOX5* expression. (Figure 8).

## Discussion

Phytosulfokine α application was verified to suppress the progression of CI degree and weight loss and the decrease in firmness and total soluble solids content (Figure 1), and to induce endogenous PSKα signaling (Figure 2), illustrating that PSKα treatment could be applied as an efficient approach to elevate tolerance to cold stress in peaches.

Phytosulfokine α treatment inhibited the gene expression of *PpLOXs* and LOX activity (Table 2 and Figures 4, 5). Low-temperature storage would cause the progression of the LOX level in postharvest fruit (Sheng et al., 2016). LOX catalyzes the hydroperoxidation of unsaturated fatty acids. Meanwhile, this process produces lots of ROS (Porta, 2002). ROS overproduction participates in the peroxidation of cell membrane lipid, resulting in cell membrane damage and cell necrosis. These physiological processes would consequently lead to browning, a typical symptom of CI. PSKα application was shown to be an effective approach to reduce O_2_^–^ production rate, H_2_O_2_ content and ROS accumulation in peaches (Figure 3), illustrating that PSKα treatment maintained redox equilibrium in peaches. Therefore, the PSKα treatment-suppressed LOX level may facilitate to alleviate redox stress, therefore relieving CI in peaches. Additionally, the gene expression of *PpCBFs* was promoted by PSKα treatment (Table 3 and Figure 6). CBFs are the clearest cold signal transduction pathway in plants (Álvaro et al., 2019). Accordingly, CBF1 promoted the expression and activity of catalase, and down regulated H_2_O_2_ content, thereby ameliorating oxidative damage and boosting chilling tolerance in transgenic tomato (Hsieh et al., 2002). Also, overexpression of *CBFc* from *Prunus mume* in Arabidopsis promoted the activity of superoxide dismutase and peroxidase, thereby boosting cold resistance (Peng et al., 2016). Therefore, the PSKα application-boosted gene expression of *PpCBFs* (Table 3 and Figure 6) may function in chilling tolerance through avoiding ROS overproduction (Figure 3). In a word, this work proved that PSKα application regulated the ROS level by weakening the gene expression of *PpLOX5* and LOX activity and boosting the gene expression of *PpCBF6*, and thus inhibited CI in peaches (Figures 1**–**6). which is consistent with a previous report suggesting that PSKα treatment enhanced the ROS scavenging capacity by elevating the expression of alternative oxidase and uncoupling protein in broccoli florets (Aghdam and Luo, 2021b). Thus, this work would provide new evidences to prove that PSKα application is an effective approach to maintain redox equilibrium in fruits and vegetables easy to suffer CI, and expand our horizon regarding the effects of PSKα on suppression of CI.

**FIGURE 9:**
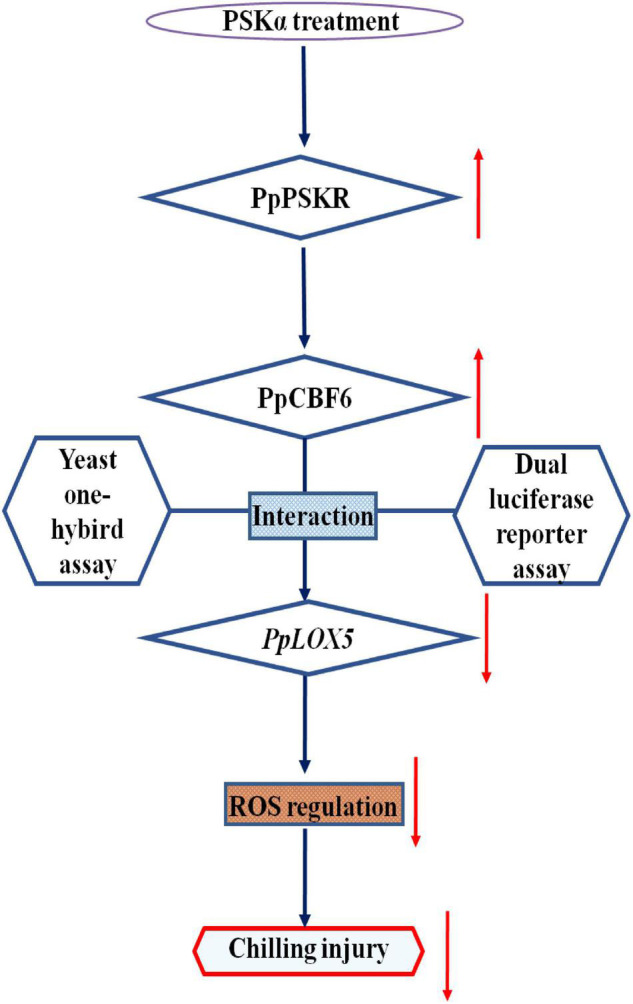
Proposed model of the involvement of PpCBF6 in the PSKα application-retarded CI by inhibiting *PpLOX5* expression in peaches.

Moreover, the promotion of chilling tolerance following exogenous applications in postharvest fruit is mediated by endogenous signals (Jiao, 2021). Both of the results of transcriptome and qRT-PCR tests suggested that the down regulation of gene expression of *PpLOX5* (LOC18774870) by PSKα treatment is the lowest (Table 2 and (Figures 4, 5), and the up regulation of gene expression of *PpCBF6* (LOC18787317) by PSKα treatment is the highest (Table 3 and Figure 6). Thus, I investigated the involvement of PpCBF6 in the PSKα-suppressed *PpLOX5* in peaches afterwards. As seen from the Y1H assay, PpCBF6 recognized the CRT/DRE motif in the promoter of *PpLOX5* (Figure 7). What’s more, the negative regulation of *PpLOX5* transcription by PpCBF6 was verified using the DLR assay (Figure 8). Accordingly, a previous study revealed that NF-YC transcription factor bound to LOX3 promoter in *Arabidopsis thaliana* (Breeze, 2014). Overexpression of *EREBP1* (a APETALA2/ethylene responsive factor transcription factor) in rice elevated the expression of chloroplastic LOX (Jisha et al., 2015). This work would broaden our perceptions regarding the molecular mechanisms of the modulation of genes in membrane lipid metabolism by transcription factors. Based on the above results, it can be inferred that when the PSKα-immersed peaches were subjected to cold stress, PSKα perception in the plasma membrane by leucine-rich repeat PSKR1 and PSKR2 may be fundamental for the up regulation of gene expression of *PpCBF6*. Then, PpCBF6 bound to *PpLOX5* promoter, and weakened its transcription. This suppression retarded ROS accumulation, thus relieving CI (Figure 9). Thus, a possible novel molecular mechanism underlying the PSKα treatment-relieved CI in peaches was elucidated in this work.

In conclusion, PSKα application reduced CI degree and weight loss, and maintained the firmness and total soluble solids content in peaches. The endogenous PSKα production and gene expression of *PSKR1* and *PSKR2* were enhanced by PSKα application. The elevation of O_2_^–^ production rate, H_2_O_2_ production and ROS content was delayed by PSKα application. Moreover, PSKα application weakened the gene expression of *PpLOX5* and LOX activity. The gene expression of *PpCBF6* was promoted by PSKα application. Furthermore, PpCBF6 interacted with a *PpLOX5* promoter fragment containing the CRT/DRE motif, and inhibited its expression. Thus, PpCBF6 mediated the PSKα-retarded CI by weakening the expression of *PpLOX5* in peaches.

## Data Availability Statement

The original contributions presented in the study are included in the article/Supplementary Material, further inquiries can be directed to the corresponding author/s.

## Author Contributions

CJ: conceptualization, formal analysis, investigation, methodology, data curation, writing, supervision, project administration, and funding acquisition.

## Conflict of Interest

The author declares that the research was conducted in the absence of any commercial or financial relationships that could be construed as a potential conflict of interest.

## Publisher’s Note

All claims expressed in this article are solely those of the authors and do not necessarily represent those of their affiliated organizations, or those of the publisher, the editors and the reviewers. Any product that may be evaluated in this article, or claim that may be made by its manufacturer, is not guaranteed or endorsed by the publisher.
