# Supplementary material for: PpCBF6 Is Involved in Phytosulfokine α-Retarded Chilling Injury by Suppressing the Expression of PpLOX5 in Peach Fruit
Source: Front Plant Sci. 2022 Apr 29;13:874338. doi: 10.3389/fpls.2022.874338 (PMC9100813; doi:10.3389/fpls.2022.874338)
Supplement: Supplementary file 1 [file Data_Sheet_1.doc]

**PpCBF6 is involved in phytosulfokine α-retarded chilling injury by suppressing the expression of *PpLOX5* in peach fruit**

Caifeng Jiao *

School of Horticulture, Anhui Agricultural University, Hefei 230036, People’s Republic of China

***Corresponding Author**

Tel/Fax: 86-0551-65786168

E-mail: 291454291@qq.com

**
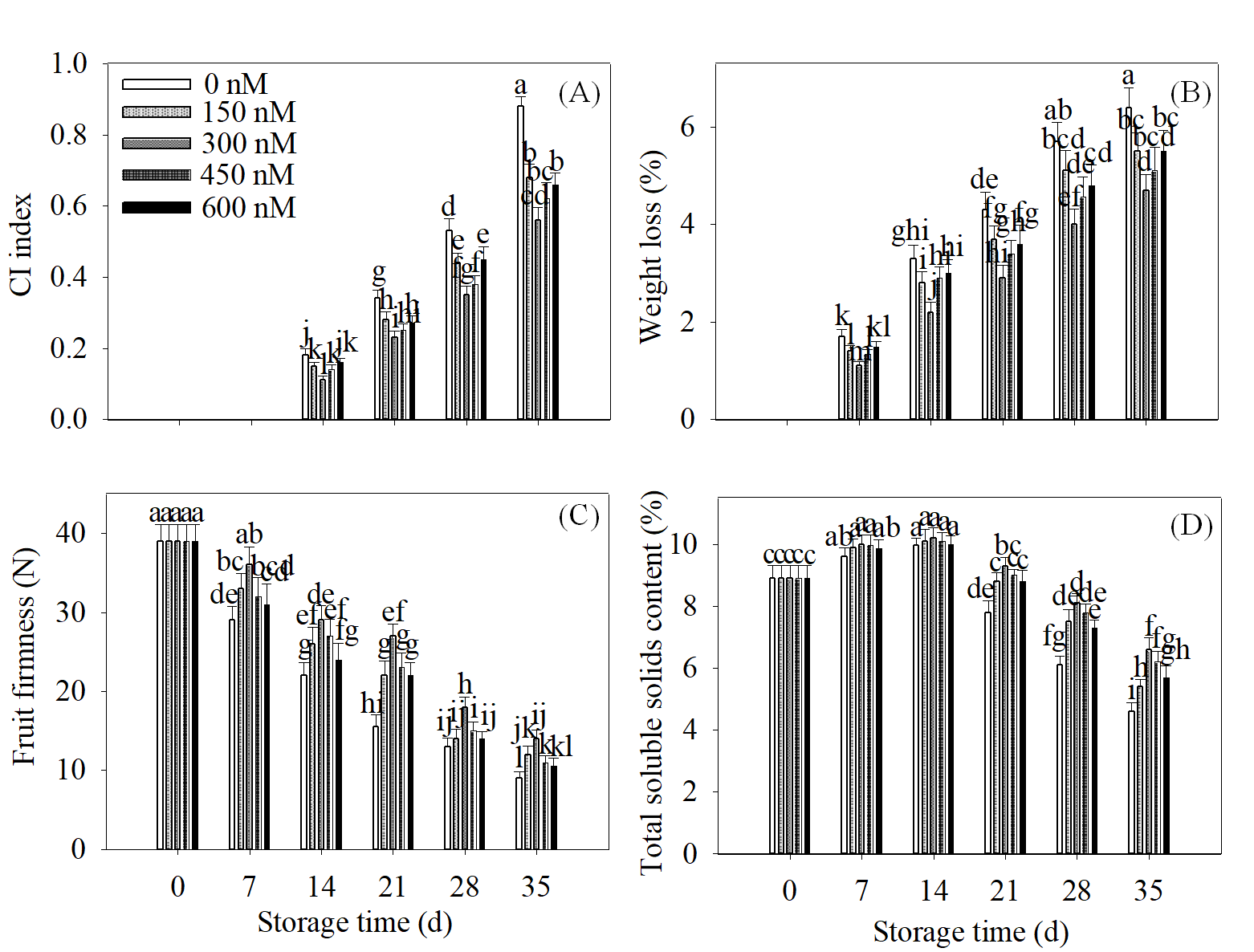
**

**Figure S1.** Effects of different concentrations of PSKα on the CI index (A), weight loss (B), firmness (C) and total soluble solids content (D) in peaches. Values represent the mean ± standard deviation. Values not with the same letter are significantly different at *p* < 0.05.

**Text S1.** Promoter nucleotide sequences of *PpLOX5*. The C-repeat/dehydration responsive element (CRT/DRE) motif (CCGAC) is marked by red. The selected sequences (the CRT/DRE motif and adjacent nucleotides) for Y1H assay are underlined. Translation start site (ATG) is shown in blue.

GCCTCTCAAGCTAGTATATAGACTAAAGCTAATTAGTATAATGCAAGTTGCAATTACTATATATAATTAAGTAGTATAATGCATTCTTTGTGTGTAATAAGCTAGAATATTACAGTTGCAATAAGGCCTTGTAAATTGCTTGGCTACAATATCTTTATATAAATAACACTGTATTCATTGGTTCATTAGCTTCATTGCATGCCTTTCAAATTCTGCAGCAACAGCCTAGGCATATGCAATATCAACATTTCATCCAAGATATTGGAAAGTATGAAAATCAGAATTTTAAGCCTTACTTTTAATAAGCATATTTGAGAGGGAGCAGCAAAAGCGTCTTTGAAATCATCCATGACAATATGCATAAGCGCCTAATCTCAATCTCAGCATGCCTGCAAATCCATAGAATAAGTTGCCTGTGTCTCTTCTGTTCAAAAATTAAATGAAAATCATCACCAAGTAAAAGCAAAAATAATATAAAATTTAAACAGTCAACTACTTCCATAAAATTAAAGCTTGAATGTTAAAATGGTCTCTGTATTTTGTATATTTGGCAAATTTAATTCATGTGTTTTTAATTTGGCCAATTTAGTTCCTGTGTTTTACTCCGTTAGCAAATGTTGTACATTATGTTAAATTACCGTTAAAAAAATTCATTAGTAACAACATTTTATATGATATTTTGAATAAAAGATTACTACTTAGGTAGGTGAAACCTTAACAATTCCAAGAAAAGAAAAAGTTCAGTCATCTTCTACCATATGGCTTGACTATAAACTCTAATGTGAAAGCTCAATATGGCTTCATCTTATCACAATATTAAAATTTAATCAGCAATTTGTTCAAGAAAAGTCATTCCAAGCACTTTCCTAGTAAATTACTTTGAAGTCTACCTACTTTACTGATTGACCTGTCATGAATGATGATGTGGCACATGGGAAACACAATTGGCTTTGGCAAGACTTTTGCAACCTCTTGAATGTAAATTCTCTAGCGTTTCCCATTGACATTATGCCCACTTCCAAAAATCTGTATATATTAATATTTTTTGGCACAGAATAACTTACGGTGTGTGGATCGAGCATAATCTGTCATTTGTGATGGGGCCAGCAAACCCTTATGTATGTATAATGTATTGGCGGTGTATTTTTTAATAACTAAATGGATAATATTAGAAAATTCAGCAAGCCATTTTTATTCAGAATCTACAGAAAGTATGTAATTGAAATCTTGCATAAATCTGTCTATATTTGTCGGTTTGGTAAAAAAAGAGGGTTGAAGCTGCCTTGAACCATCGTCATCATCATCATCAAATTCCAAAATTGACATACAACTGTAAACAATAGGAAAAGTCTGGGTCTTTTAAGTCAACCCCTGCAAATGCAATAGAACAAATAAAAAGAATTTTTATTTATTTATTGTTGGGTCATGATGAGAACAACATAAGATTGAGTTGGTGAAGTTTAAAGACCAGAACTTGAGGCCAAAAGCTGTAAAATCAGTATGACTAGGATTTCATAAAGACTGATACAGTGAGCTCACATCACACTTCCCATAAATACCACACCTTCTCTCACTAATTCTTCACACTGACTGAGATCCTCAAAACCTTCTTTTATTAAATTTTCTCAGTTCTTTCTTGAAGCAGCAGACAAAG**ATG**
